# Candidate Olfaction Genes Identified within the *Helicoverpa armigera* Antennal Transcriptome

**DOI:** 10.1371/journal.pone.0048260

**Published:** 2012-10-26

**Authors:** Yang Liu, Shaohua Gu, Yongjun Zhang, Yuyuan Guo, Guirong Wang

**Affiliations:** State Key Laboratory for Biology of Plant Diseases and Insect Pests, Institute of Plant Protection, Chinese Academy of Agricultural Sciences, Beijing, China; Duke University, United States of America

## Abstract

Antennal olfaction is extremely important for insect survival, mediating key behaviors such as host preference, mate choice, and oviposition site selection. Multiple antennal proteins are involved in olfactory signal transduction pathways. Of these, odorant receptors (ORs) and ionotropic receptors (IRs) confer specificity on olfactory sensory neuron responses. In this study, we identified the olfactory gene repertoire of the economically important agricultural pest moth, *Helicoverpa armigera*, by assembling the adult male and female antennal transcriptomes. Within the male and female antennal transcriptomes we identified a total of 47 OR candidate genes containing 6 pheromone receptor candidates. Additionally, 12 IR genes as well as 26 odorant-binding proteins and 12 chemosensory proteins were annotated. Our results allow a systematic functional analysis across much of conventional ORs repertoire and newly reported IRs mediating the key olfaction-mediated behaviors of *H. armigera*.

## Introduction

Olfaction, the sense of smell, plays a predominant role in mediating insect behavior including food source identification, oviposition site selection, mate choice, kin recognition and predator avoidance. Of insect olfactory events, sexual communication in moths is an excellent model system for understanding the mechanism of animal sensory perception at the molecular level because of the complexity, specificity and extreme sensitivity of males to specific pheromone molecules emitted from conspecific females [1], [2].

The surface of insect antennae is covered with several different types of sensilla that are small sensory hair structures in which olfactory receptor neurons extend dendrites into the antennal lymph where peripheral olfactory signal transduction events occur. Previous studies have shown diverse olfactory genes including at least odorant-binding proteins (OBPs), chemosensory proteins (CSPs), Sensory neuron membrane proteins (SNMPs), odorant-degrading enzymes (ODEs), ionotropic receptors (IRs) and odorant receptors (ORs) involved in different steps in signal transduction pathway [3], [4], [5]. All of these, ORs play a central role in chemosensory signal transduction processes that occur in olfactory receptor neurons. ORs located on the surface of olfactory sensory neuronal dendrites in the antennae possess seven transmembrane domains (TMDs). In insects, it is generally thought that odor recognition is mediated by a single set of odor receptor heterodimers composed of a conserved, nonconventional ORCO protein acting as an ion channel and a variable, conventional OR that apparently mediates odorant-binding specificity [6], [7], [8], [9], [10], [11]. In addition, a novel family of candidate chemosensory ionotropic receptors (IRs) involved in odorant recognition was recently characterized by a genome-based bioinformatics screen in *Drosophila melanogaster* [12]. IRs are not closely related to ionotropic glutamate receptors in the phylogenetic analysis. However, they possess an obviously similar modular organization to ionotropic glutamate receptors [12], [13]. Like ORs, IRs are extremely divergent and expresssed in sensory dendrites. Misexpression of several Drosophila IRs suggested that they might be tuned to a small odor panel such as small amine-like volatile compounds [12], [14], [15].

In addition, the aforementioned multiple proteins may interact with ORs/IRs and are required for olfactory signal transduction. The soluble OBPs are thought to facilitate the transport of odorant molecules through the sensillum lymph and are sometimes thought to be directly involved in ligand binding [16], [17], [18]. The CSPs are another class of soluble proteins in the sensillum lymph with abundant expression, however their function in olfactory transduction remains largely unknown [16], [19]. SNMPs locate in the dendritic membrane of peripherally olfactory receptor neurons just adjoining ORs and are presumed to trigger ligand delivery to the receptor [20], [21], [22]. Within the olfactory sensilla, ODEs are reputedly involved in the signal inactivation step serving to rapidly remove stimulatory odorant molecules [23], [24].

Genome sequencing and molecular studies have together characterized the complete OR repertoires and other olfactory genes in several insect species such as *D. melanogaster*, *Anopheles gambiae*, *Bombyx mori* and others, promoting understanding of olfactory signal pathways in these insects. However, a systematic analysis of the olfactory genes especially ORs/IRs is still largely absent in from most insect species, including important crop pest insects where a genome sequence is not yet unavailable. Traditional homology-based cloning strategies could identify some conserved genes such as ORCO and OBPs, but would not readily identify divergent genes, especially the divergent ORs and IRs families. Therefore, recent studies of the molecular mechanisms of olfactory signal transduction in moths have largely been limited to *B. mori* due to the availability of genome sequence. In other Lepidoptera species, only a few ORs and PRs have been identified [25], [26], [27], [28], [29], [30], [31]. Expressed Sequence Tag (EST) sequencing strategies have been successfully used to identify olfactory genes in Lepidoptera without genomic data such as *Manduca sexta* [32], *Epiphyas postvittana* [33] and *Spodoptera littoralis* [34], [35]. But most identified genes are OBPs; relatively few are divergent genes such as ORs and IRs. The identification of moth ORs and IRs is still a challenging exercise. Recently, RNA-seq approaches have been used to identify olfactory genes in species where a genome sequence is not yet available. To date, next-generation sequencing of antennal transcriptome has been successfully used to identify substantial numbers of candidate ORs and IRs in *M. sexta* [36], *Cydia pomonella* [37], and *Bactrocera dorsalis* [38].

Moths are a diverse group of insects that include many economically important crop pests. The subfamily heliothinae contains approximately 365 species of noctuid moths including the world’s most injurious crop pests such as the Old World bollworm and relatives, and the New World tobacco budworm, which cause huge economic loss annually [39], [40], [41]. Better understanding on the molecular mechanisms of olfactory recognition in these moths could ultimately lead to identify new targets for developing environment-friendly control strategies.

To explore the olfaction-related transcriptome of the worldwide agricultural pest, *H. armigera*, we conducted 454 pyrosequencing of RNA extracted from adult male and female antennae. Our goals were to identify olfaction-related genes and olfactory signal transduction mechanisms in *H. armigera* antennae. Here we report the identification of 47 candidate OR genes and 12 IR genes as well as 26 OBP genes and 12 CSP genes in the male and female antennal transcriptomes.

## Methods

### Insect Rearing

*Helicoverpa armigera* used in all experiments were obtained from a colony maintained at the Institute of Plant Protection, Chinese Academy of Agricultural Sciences, Beijing, China. Larvae were reared on an artificial diet at 27±1°C under a photoperiod of 14∶10 (L: D). Male and female pupae were placed in separate glass tubes. Adults were fed 10% honey solution. Antennae of female or male individuals were dissected 1–3 days after eclosion and immediately frozen in liquid nitrogen, and stored at –70°C until extraction.

### Extraction of Total RNA

Frozen antennae were transferred to a liquid nitrogen cooled mortar and ground with a pestle. The homogenate was covered with 1 mL of TriZol reagent (Invitrogen, Carlsbad, CA, USA) and total RNA extractions were performed following the manufacturer’s instructions. Total RNA was dissolved in H_2_O and RNA integrity was verified by gel electrophoresis. RNA quantity was determined on a Nanodrop ND-1000 spectrophotometer (NanoDrop products, Wilmington, DE, USA).

### Sequencing

PolyA+mRNA was separated from 10 µg of total RNA extracted from approximately 300 antennae of 1–3 day old adult male or female moths using the PolyA+ Ttract mRNA Isolation System (Promega, Madison, WI, USA) and further purified by using the RNeasy MinElute Clean up Kit (Qiagen, Hilden, Germany) following the manufacturer’s protocol. mRNA integrity and quantity were verified on an Agilent 2100 Bioanalyzer. mRNA was fragmented in the RNA fragment reagent for 1 min according to the NEBNext® Magnesium RNA Fragmentation Module Protocol (NEB, Ipswich, MA, USA). After recovered by RNeasy MinElute Clean up Kit, the fragmented mRNA larger than 250 bp was used to synthesize double-stranded–cDNA (ds-cDNA). The first strand cDNA was synthesized using random primers and MMLV reverse transcriptase (Promega, Madison, WI, USA). The second strand was then synthesized using *E. coli* DNA Polymerase I and RNase H. The resulting ds-cDNAs were used as a template for NextGen sequencing on a Roche 454 FLX by using standard chemistry according to the manufacturer's instructions (Roche, Branford, CT, USA). One full picotiterplate (PTP) with two regions was used for sequencing the male and female samples. 1/2 run data of each sample was generated separately for next analysis.

### Unigene Generation

The raw 454 sequences in SFF files were extracted using the Python script sff_extract.py developed by COMAV (http://bioinf.comav.upv.es). All the raw sequences were then processed to remove low quality and adaptor sequences using programs TAGDUST [42], LUCY [43] and SeqClean [44]. The resulting sequences were then screened against the NCBI UniVec database and bacterial genome sequences to remove possible contaminants. Sequences shorter than 50 bp were discarded. The cleaned 454 read sequences from both males and females were assembled into a single assembly by Newbler version 2.5.3 using default parameters under the cDNA option (Roche, Branford, CT, USA).After assembly, the clean reads of both male and females samples were mapped to the assembled unigenes using GS Reference Mapper (Roche, Branford, CT, USA). These reads in male and female samples mapped to each unigene were derived and counted separately. If all the reads mapped certain unigene were from male or female samples, this unigene was determined as male or female special.

### Gene Identification and Functional Annotation

The unigenes were searched against the NCBI non-redundant protein database on a local server using the National Center for Biotechnology Information (NCBI) BLASTALL program [45]. GO Annotation was performed by using Blast2GO (GO association done by a BLASTX against the NCBI NR database) [46], [47]. The ORFs of the unigenes were predicted by using ORF finder (http://www.ncbi.nlm.nih.gov/gorf/gorf.html). The signal peptide of the protein sequences were predicted using SignalP 4.0 [48]. The TMDs of annotated genes were predicted using TMHMM Server Version2.0 (http://www.cbs.dtu.dk/services/TMHMM). *H. armigera* antennal unigenes were searched with *B. mori* ORs [49], OBPs [50] and IRs [15] as queries using TBLASTN in the free software BioEdit program. Read numbers of each unigene in male and female samples were derived and counted separately.

### Phylogenetic Analyses

The phylogenetic reconstruction implemented for the analysis of OR, IR, OBP and CSP was performed based on the amino sequences of the candidate olfaction genes and the collected data sets. The OR data set contained OR sequences identified in Lepidoptera (12 from *H. armigera*, 21 from *H. virescens* and 64 from *B. mori*) [29], [30], [49], [51], [52]. The IR data set contained 12, 18 and 66 IR sequences from *S. littoralis*, *B. mori* and *D. melanogaster*, respectively [15], [35]. The OBP data set contained 15 sequences from *H. armigera*, 17 sequences from *H. virescens* and 35 sequences from *B. mori* [50], [53]. The CSP data set contained the 7 sequences from *H. armigera* [53], 9 sequences from *H. virescens* [54], two sequences from *Spodoptera exigua* and the 16 sequences from *B. mori* [19]. The protein name and accession number of the genes used for phylogenetic tree building are listed in supplementary material S1. Amino acid sequences were aligned using ClustalW2 [55]. Unrooted trees were constructed by the neighbor-joining method, with Poisson correction of distances, as implemented in MEGA5 software [56]. Node support was assessed using a bootstrap procedure base on 1000 replicates.

### Expression Analysis of the Candidate Receptors by Semi-quantitative Reverse Transcription PCR

To illustrate and compare the expression of candidate receptors in male and female antennae, semi-quantitative reverse transcription PCR was performed using cDNAs prepared from male antennae, female antennae and legs (male and female mixture). Legs were used as a control to verify that the candidate receptors were antennae enriched. Total RNAs were extracted as described above. Prior to cDNA synthesis, RNA were treated with DNase I (Fermentas, Vilnius, Lithuania) to remove trace amounts of genomic DNA. The cDNA was synthesized by First Strand cDNA Synthesis Kit (Fermentas, Vilnius, Lithuania) and was used as a template in PCR reactions with gene-specific primers. An actin gene fragment was used as control. Primers were designed using the Primer Premier 5 software (PREMIER Biosoft International). The primer sequences are available in supplementary material S2. PCR was performed with Veriti Thermal Cycler (Applied Biosystems, Carlsbad, CA, USA) under the following conditions: 94°C for 2 min, 33 cycles of 94°C for 30 s, 55–60°C for 30 s; 72°C for 30 s, and 72°C for 8 min. The cycle number was reduced to 26 and 29 for Actin and OR2 amplifications because of their high expression level. The experiment was repeated three times using three independently isolated RNA samples. PCR amplification products were run on a 2% agarose gel and verified by DNA sequencing.

## Results

### Sequencing and Unigene Assembly

Using GS/FLX 454 pyrosequencing technology, a total of 753,643 raw reads were obtained from the male sample and 518,746 raw reads from female sample. After removing low quality, adaptor, and contaminating sequence reads, male and female antennae yielded 731,001 (average read length 522 bp) and 463,908 (average read length 431 bp) clean reads, respectively. The total bases of sequence data were approximately 382 million and 200 million from male and female samples, respectively. All clean reads from male and female samples were combined into an assembly that generated 37,920 unigenes larger than 100 bp with 991 bp average length. Of these, 8,706 (23.0%) unigenes were from the male antennae and 3,698 (9.8%) unigenes were from female antennae. A flow chart of sequencing and unigene assembly are shown in supplementary material S3. The gene length, ORF length, read number in male or female samples of each unigene were integrated in supplement material S4.

### Gene Identification and Functional Annotation

**Figure 1:**
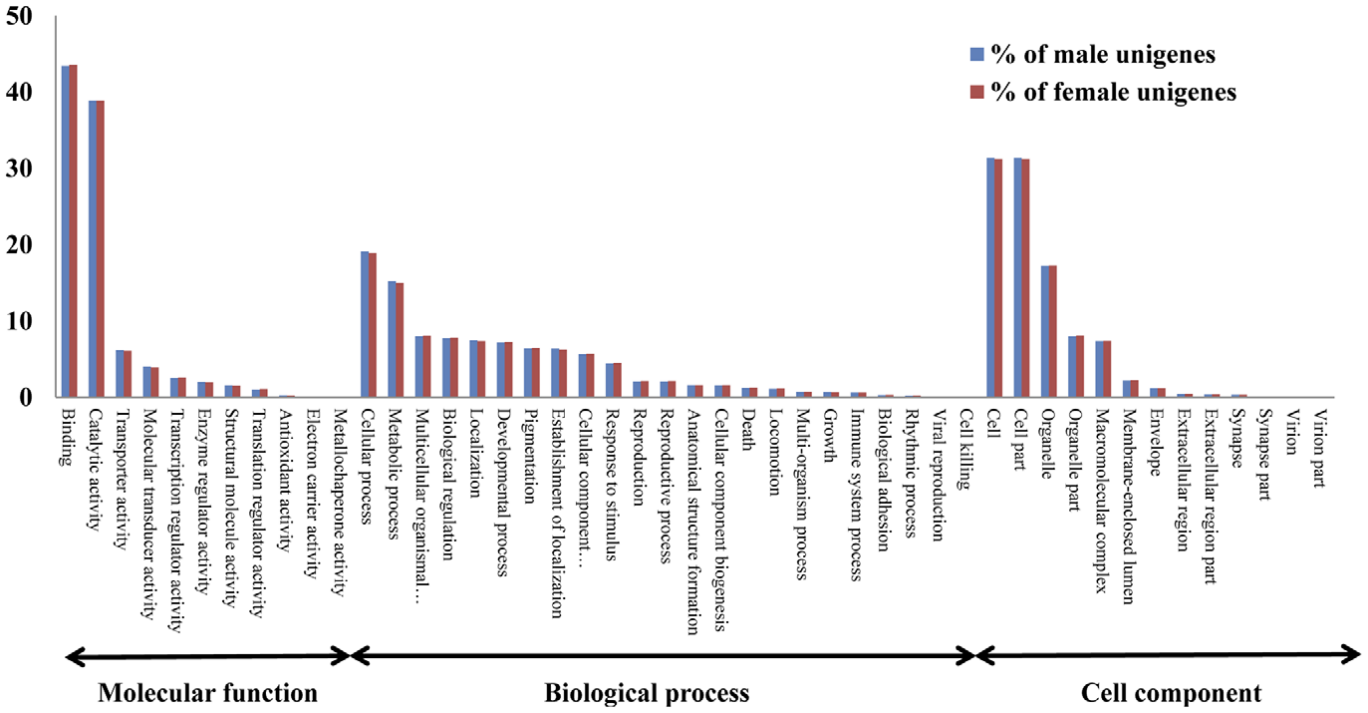
Distribution and comparison of male and female *H. armigera* unigenes annotated at GO level 2. The Y-axis shows the percentage of the sequences. The X-axis shows three areas of annotation, and in each area the sequences are further divided into subgroups at GO level 2.

The gene functional annotation was first performed by GO annotation using Blast2GO. Figure 1 illustrates the distribution of the unigene set in GO terms. Among the 37,920 unigenes, 11,233 (29.6%) corresponded to at least one GO term (Figure 1), 9,164 were assigned to a molecular function (24.2%), 8,329 to a biological processes (22%), and 6,588 to a cellular component (17.4%). There was no difference between the GO terms of male and female sets. In the molecular function category, binding and catalytic activities were the most abundant and enriched GOs terms in both male and female sets. In the biological process terms, cellular and metabolic processes were the most represented. In the cellular component terms, cell, cell part and organelle were the most abundant (Figure 1). GO annotations of the unigenes are presented in supplementary material S4.

The unigenes were then searched against the NCBI non-redundant nucleotide and protein database using BLASTN and BLASTX. Of the 37,920 unigenes, 24,675 (65.1%) showed similarity to known proteins supplementary material S1). The ORs, IRs, OBPs and CSPs were annotated according to the BLAST result. The BLASTN and BLASTX best hit result is listed in supplementary material S4.

### Identification of Candidate Chemosensory Receptors

**Figure 2:**
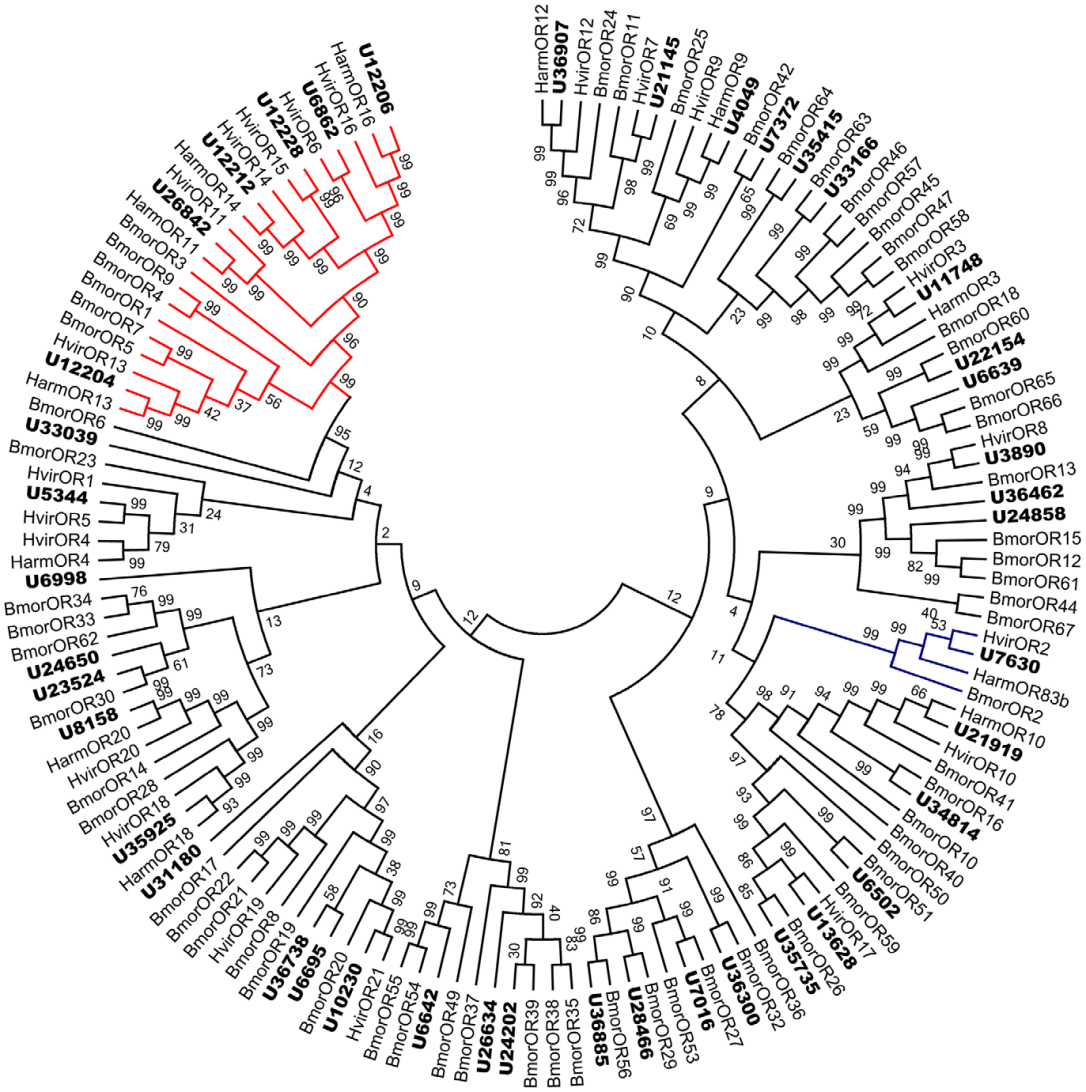
Phylogenetic tree of candidate ORs from lepidopterans including PR (red) and ORCO (Blue) clades. Harm: *H. armigera*, Hvir: *H. virescens* ***,*** Bmor: *B. mori*. The *H. armigera* unigenes are shown in bold and the letter-unigene in the unigene reference was abbreviated as U.

All the contigs were searched by BLASTX and further by TBLASTN using 63 and 21 known ORs from *B. mori* and *H. virescens,* respectively, leading to identification of 47 different contigs that were putative OR genes. All 47 sequences possessed overlapping regions with low identity, and therefore, likely represent unigenes. Of these, 13 HarmOR sequences had full-length open reading frames (ORF) with 5–6 transmembrane domains characteristic of typical insect ORs. Not surprisingly, a HarmOR sequence that shared very high identity (∼up to 90%) with the conserved insect co-receptor was identified. As other insect ORs, most HarmORs were highly divergent and shared low similarity with other insect ORs except for closely related species such as *H. virescens*. However, six HarmORs shared considerable similarity and were classified into a subgroup in the phylogenetic tree with previously characterized lepidopteran pheromone receptors (Figure 2). Almost all odorant receptor candidates were clustered with at least one lepidopteran orthologous gene in the phylogenetic tree except for two ORs (unigene6998 and 31180) appear to be more distant from their closest homolog because of their short length. We named the candidate OR unigenes according to their similarity to known ORs. The unigenes which had high similarity to *H. virescens* ORs were named following their orthologous ORs. Unigene6695 was named as HarmOR21.2 because of its relatively low similarity to HvirOR21. The remaining 27 ORs were named HarmOR22 through HarmOR48. The information including unigene reference, length, BLASTx best hit of all 47 ORs and 1 GR are listed in Table 1. The nucleotide sequences of all 47 ORs and 1 GR are listed in supplementary material S5.

**Table 1:** Unigenes of candidate olfactory receptors and gustatory receptor.

Unigene reference	Gene name	Length (bp)	ORF (aa)	BLASTx best hit (Reference/Name/Species)	E value	Identity	Full length	TMD (No)	Sexual specificity
**Co-receptor**									
unigene7630	HarmOR2	2588	473	gb|ADQ13177.1| olfactory receptor OR83b [Helicoverpa armigera]	0.0	99%	Yes	7	No
**Pheromone receptors**									
unigene26842	HarmOR11	1736	430	gb|ACS45305.1| candidate odorant receptor 2 [Helicoverpa armigera]	0.0	99%	Yes	6	No
unigene12204	HarmOR13	1596	425	gb|ACS45304.1| candidate odorant receptor 1 [Helicoverpa armigera]	0.0	99%	Yes	7	Male
unigene12212	HarmOR14	1378	313	gb|ACF32964.1| olfactory receptor 14 [Helicoverpa armigera]	0.0	99%	No	4	Male
unigene12228	HarmOR15	1231	387	emb|CAG38116.1| putative chemosensory receptor 15 [Heliothis virescens]	0.0	86%	No	4	Male
unigene12206	HarmOR16	1616	422	gb|ACS45306.1| candidate odorant receptor 3 [Helicoverpa armigera]	0.0	97%	Yes	3	Male
unigene6862	HarmOR6	719	191	emb|CAD31948.1| putative chemosensory receptor 6 [Heliothis virescens]	1e−103	85%	No	0	No
**Olfactory receptors**									
unigene11748	HarmOR3	699	200	emb|CAD31852.1| putative chemosensory receptor 3 [Heliothis virescens]	6e−129	94%	No	2	No
unigene5344	HarmOR5	430	142	emb|CAD31947.1| putative chemosensory receptor 5 [Heliothis virescens]	3e−93	96%	No	2	No
unigene21145	HarmOR7	814	271	emb|CAD31853.1| putative chemosensory receptor 7 [Heliothis virescens]	1e−172	96%	No	4	No
unigene3890	HarmOR8	1331	351	emb|CAD31949.1| putative chemosensory receptor 8 [Heliothis virescens]	0.0	73%	No	6	No
unigene4049	HarmOR9	764	243	emb|CAD31950.1| putative chemosensory receptor 9 [Heliothis virescens]	2e−140	86%	No	4	No
unigene21919	HarmOR10	666	221	gb|ACC63238.1| olfactory receptor 10 [Helicoverpa armigera]	0.0	100%	No	3	No
unigene36907	HarmOR12	1368	408	gb|ACF32963.1| olfactory receptor 12 [Helicoverpa armigera]	0.0	97%	Yes	6	No
unigene13628	HarmOR17	1339	396	emb|CAG38118.1| putative chemosensory receptor 17 [Heliothis virescens]	0.0	93%	Yes	6	Male
Unigene35925	HarmOR18	1356	398	gb|ACL81187.1| putative olfactory receptor 18 [Helicoverpa armigera]	0	100%	Yes	5	No
unigene186	HarmOR19	429	142	emb|CAG38120.1| putative chemosensory receptor 19 [Heliothis virescens]	5e−49	61%	No	1	Female
unigene8158	HarmOR20	1269	387	gb|ACC63240.1| olfactory receptor 20, partial [Helicoverpa armigera]	0	99%	Yes	7	No
unigene10230	HarmOR21	939	278	emb|CAG38122.1| putative chemosensory receptor 21 [Heliothis virescens]	2e−160	85%	No	5	No
unigene6695	HarmOR21.2	1237	396	emb|CAG38122.1| putative chemosensory receptor 21 [Heliothis virescens]	2e−86	37%	No	6	No
unigene36738	HarmOR22	1040	346	gb|ADM32898.1| odorant receptor OR-5 [Manduca sexta]	6e−54	36%	No	4	No
unigene6998	HarmOR23	411	135	gb|AEF32141.1| odorant receptor [Spodoptera exigua]	2e−63	73%	No	2	No
unigene35735	HarmOR24	1197	358	dbj|BAF31195.1| candidate olfactory receptor [Bombyx mori]	7e−151	67%	No	4	No
unigene24858	HarmOR25	1167	389	tpg|DAA05974.1| TPA_exp: odorant receptor 15 [Bombyx mori]	4e−115	48%	No	4	No
unigene6502	HarmOR26	693	212	dbj|BAH66346.1| olfactory receptor [Bombyx mori]	5e−95	67%	No	2	No
unigene36300	HarmOR27	2136	413	dbj|BAH66328.1| olfactory receptor [Bombyx mori]	3e−108	51%	Yes	6	No
unigene24202	HarmOR28	671	185	dbj|BAH66335.1| olfactory receptor [Bombyx mori]	3e−54	57%	No	2	No
unigene36462	HarmOR29	1619	395	ref|NP_001166603.1| olfactory receptor 13 [Bombyx mori]	7e−127	48%	Yes	7	No
unigene34814	HarmOR30	703	229	ref|NP_001104832.2| olfactory receptor 16 [Bombyx mori]	2e−111	70%	No	4	No
unigene28466	HarmOR31	975	325	ref|NP_001166894.1| olfactory receptor 29 [Bombyx mori]	2e−160	71%	No	6	No
unigene24650	HarmOR32	1069	349	ref|NP_001103623.1| olfactory receptor 33 [Bombyx mori]	2e−99	42%	No	3	No
unigene26634	HarmOR33	1195	398	ref|NP_001103476.1| olfactory receptor 35 [Bombyx mori]	4e−138	51%	No	4	No
unigene30070	HarmOR34	550	183	tpg|DAA05992.1| TPA_exp: odorant receptor 36 [Bombyx mori]	8e−69	53%	No	3	No
unigene7372	HarmOR35	867	241	ref|NP_001091818.1| olfactory receptor 42 [Bombyx mori]	3e−27	31%	No	3	No
unigene31113	HarmOR36	321	106	ref|NP_001091818.1| olfactory receptor 42 [Bombyx mori]	6e−38	58%	No	2	No
unigene14199	HarmOR37	455	136	ref|NP_001166607.1| olfactory receptor 44 [Bombyx mori]	7e−78	86%	No	2	Male
unigene31180	HarmOR38	428	142	ref|NP_001166607.1| olfactory receptor 44 [Bombyx mori]	1e−23	75%	No	1	No
unigene6642	HarmOR39	1272	377	ref|NP_001166616.1| olfactory receptor 54 [Bombyx mori]	9e−73	38%	No	6	No
unigene7016	HarmOR40	910	303	ref|NP_001166893.1| olfactory receptor 27 [Bombyx mori]	8e−113	63%	No	5	No
unigene36885	HarmOR41	1803	179	ref|NP_001166617.1| olfactory receptor 56 [Bombyx mori]	1e−87	78%	No	3	No
unigene22154	HarmOR42	1237	392	ref|NP_001155301.1| olfactory receptor 60 [Bombyx mori]	0	70%	No	5	No
unigene33166	HarmOR43	1247	393	ref|NP_001166620.1| olfactory receptor 63 [Bombyx mori]	6e−132	52%	Yes	7	No
unigene35415	HarmOR44	1245	375	ref|NP_001166621.1| olfactory receptor 64 [Bombyx mori]	7e−91	53%	Yes	7	No
unigene6639	HarmOR45	1513	434	ref|NP_001166622.1| olfactory receptor 65 [Bombyx mori]	2e−79	63%	Yes	7	Female
unigene33039	HarmOR46	1041	346	ref|NP_001116817.1| olfactory receptor-like [Bombyx mori]	3e−147	69%	No	5	No
unigene5523	HarmOR47	320	106	tpg|DAA05974.1| TPA_exp: odorant receptor 15 [Bombyx mori]	8e−22	40%	No	1	No
unigene23524	HarmOR48	639	197	tpg|DAA05986.1| TPA_exp: odorant receptor 30 [Bombyx mori]	1e−73	54%	No	1	No
**Gustatory receptors**									
unigene14001	HarmGR1	632	210	tpg|DAA06395.1| TPA_inf: gustatory receptor 63 [Bombyx mori]	1e−49	47%	No		No

### Identification of Candidate Ionotropic Receptors

**Figure 3:**
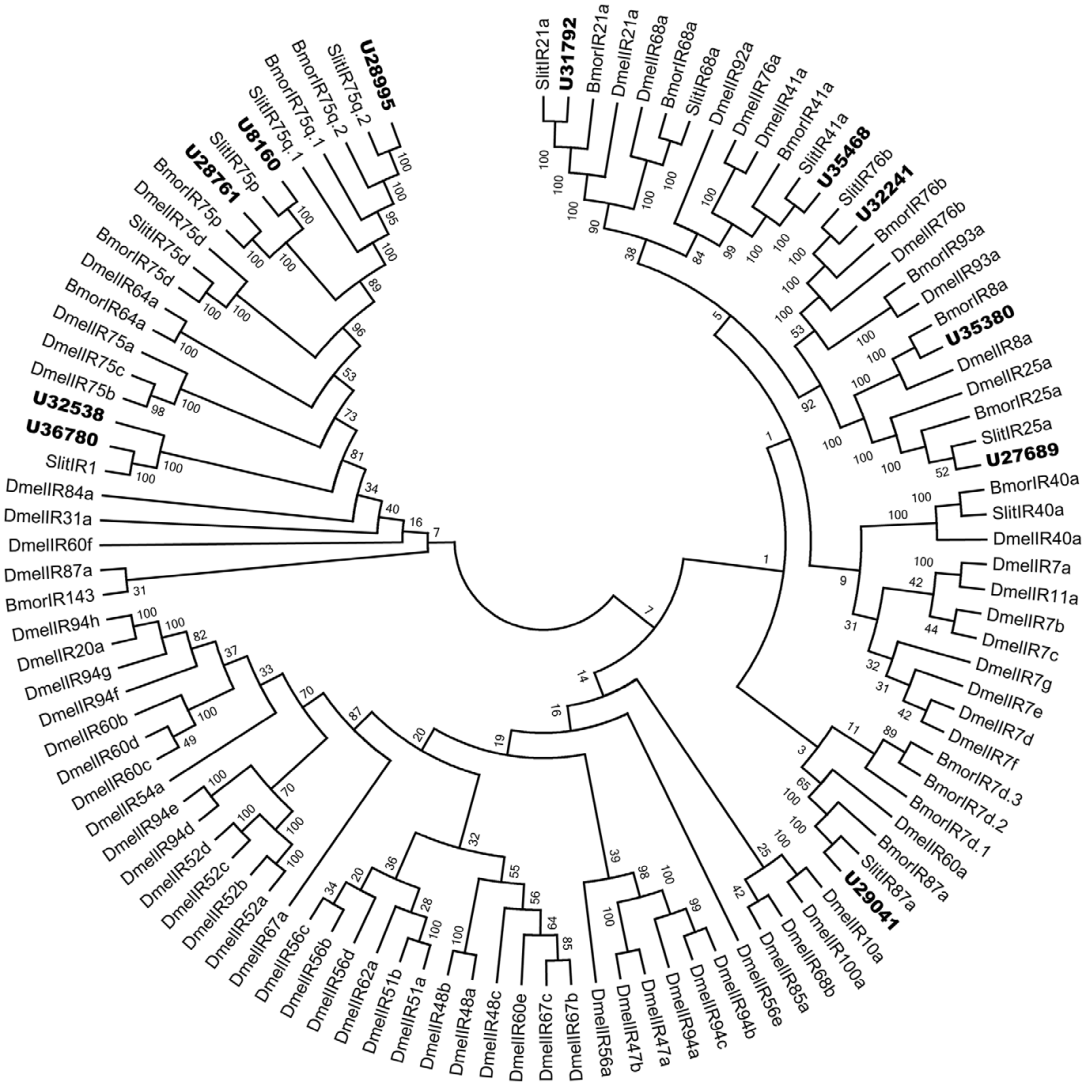
Phylogenetic tree of candidate IRs from insects. Slit: *S. littoralis*, Bmor: *B. mori*, Dmel: *D. melanogaster*, the *H. armigera* unigenes are shown in bold and the letter-unigene in the unigene reference was abbreviated as U.

The IRs sequences in the *H. armigera* antennal transcriptome assembly were represented according to the similarity with known insect IRs. Bioinformatic analysis led to the identification of 12 candidates IRs. Sequence analysis identified 9 unigenes with a full length ORF. The insect IRs contained three transmembrane domains. TMHMM2.0 predicted 11 candidate IRs with three transmembrane domains (Table 2). Eight of the 12 putative IRs had at least 68% identity with the corresponding IRs of *S. littoralis*. These may be the orthologous genes in *H. armigera*. One candidate IR was represented to be IR8a due to its high identity to BmorIR8a. The remaining three putative IRs had relatively low similarity to other insect IRs. Unigene27689 had 65% identity with IR25a of *D. melanogaster*. Unigene32538 had 34% identity with IR1 of *S. littoralis*. Unigene28761 had 61% identity with IR75p of *S. littoralis*. The orthologs of these genes probably haven’t been identified in *S. littoralis*. The phylogenetic analyses validated accurate prediction of the IRs. In the neighbor-joining tree of IRs, all candidate *H. armigera* IRs clustered with their orthologs of *S. littoralis* and *B. mori* into a separate clade (Figure 3). Eleven of the 12 candidate IR unigenes were named according to their similarity to known IRs. The new IR unigene32538 was named HarmIR1.2. The information including unigene reference, length, and BLASTx best hit of all the12 IRs are listed in Table 2. The nucleotide sequences of all 12 IRs were listed in supplementary material S5.

**Table 2:** Unigenes of candidate ionotropic receptors.

Unigene reference	Gene name	Length (bp)	ORF (aa)	Blastx best hit (Reference/Name/Species)	E value	Identity	Full length	TMD (No)
unigene36780	HarmIR1	2070	617	gb|ADR64688.1| putative chemosensory ionotropic receptor IR1 [Spodoptera littoralis]	0.0	68%	Yes	3
unigene32538	HarmIR1.2	1862	620	gb|ADR64688.1| putative chemosensory ionotropic receptor IR1 [Spodoptera littoralis]	4e−42	34%	Yes	2
unigene35380	HarmIR8a	3456	895	gb|AFC91764.1| putative ionotropic receptor IR8a, partial [Cydia pomonella]	0.0	81%	Yes	3
unigene31792	HarmIR21a	2927	857	gb|ADR64678.1| putative chemosensory ionotropic receptor IR21a [Spodoptera littoralis]	0.0	82%	Yes	3
unigene27689	HarmIR25a	2966	918	gb|AFC91757.1| putative ionotropic receptor IR25a [Cydia pomonella]	0.0	87%	Yes	3
unigene35468	HarmIR41a	2014	608	gb|ADR64681.1| putative chemosensory ionotropic receptor IR41a [Spodoptera littoralis]	0.0	81%	Yes	3
unigene21502	HarmIR75d	840	231	gb|ADR64683.1| putative chemosensory ionotropic receptor IR75d [Spodoptera littoralis]	1e−71	81%	No	3
unigene8160	HarmIR75p	1681	547	gb|ADR64684.1| putative chemosensory ionotropic receptor IR75p [Spodoptera littoralis]	0.0	90%	No	3
unigene28761	HarmIR75p.2	2368	654	gb|ADR64684.1| putative chemosensory ionotropic receptor IR75p [Spodoptera littoralis]	0.0	61%	Yes	3
unigene28995	HarmIR75q.2	4238	628	gb|ADR64685.1| putative chemosensory ionotropic receptor IR75q.2 [Spodoptera littoralis]	0.0	83%	No	3
unigene32241	HarmIR76b	1879	558	gb|ADR64687.1| putative chemosensory ionotropic receptor IR76b [Spodoptera littoralis]	0.0	84%	Yes	3
unigene29041	HarmIR87a	1919	583	gb|ADR64689.1| putative chemosensory ionotropic receptor IR87a [Spodoptera littoralis]	0.0	94%	Yes	3

### Identification of Putative Odorant-binding Proteins

**Figure 4:**
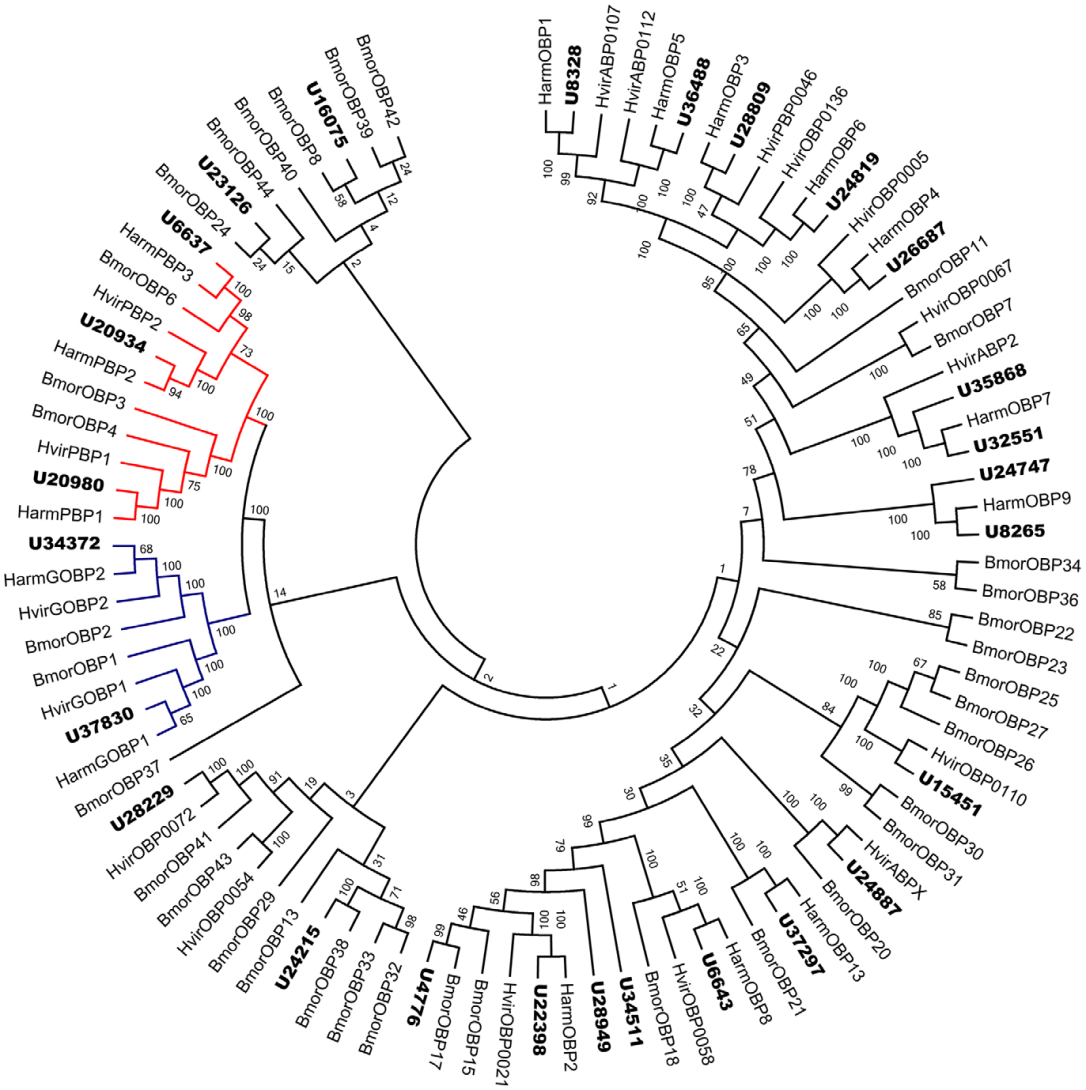
Phylogenetic tree of candidate odorant binding protein from lepidopterans including PBP (red), GOBP (Blue) and other OBP. Harm: *H. armigera*, Hvir: *H. virescens,* Bmor: *B. mori*, Dple: *D. plexippus*, the *H. armigera* unigenes are shown in bold and the letter-unigene in the unigene reference was abbreviated as U.

Within the *H. armigera* antennal transcriptome 26 different sequences encoding odorant binding proteins were identified, including three PBPs and two GOBPs. Sequence analysis identified 19 unigenes with a full length ORF with a predicted signal peptide sequence. Signal peptide sequence was not detected in the remainder of putative OBPs due to incomplete N-termini. All 26 putative OBPs had high similarity to known lepidopteran OBPs. Unigene35868 and unigene24747 had very high similarity with HarmOBP7 and HarmOBP9 (94% and 95%, respectively). They may be isoforms of the respective *H. armigera* OBPs or potentially new OBP genes. As expected, the PBP and GOBP sequences were clustered in separate clade in the OBP neighbor-joining tree. All the candidate OBP sequences were clustered with at least one lepidopteran ortholog, in congruence with the BLAST results (Figure 4). HarmOBPs were named according to their similarities with previously annotated *H. armigera* OBPs. Two of the putative OBP isoforms, unigene35868 and unigene24747, were named as HarmOBP7.2 and HarmOBP9.2, respectively. The information including unigene reference, length, and BLASTx best hit and so on of all the 26 OBPs was listed in table 3. The nucleotide sequences of all the 26 OBPs were listed in supplement material S5.

**Table 3:** Unigenes of candidate odorant binding proteins.

Unigene reference	Gene name	Length (bp)	ORF (aa)	Blastx best hit (Reference/Name/Species)	E value	Identity	Full length	Signal peptide
**Pheromone binding protein**								
unigene20980	HarmPBP1	1253	170	gb|AEB54585.1| PBP1 [Helicoverpa armigera]	2e−108	99%	Yes	Yes
unigene20934	HarmPBP2	989	165	gb|AEB54583.1| PBP2 [Helicoverpa armigera]	2e−114	98%	Yes	Yes
unigene6637	HarmPBP3	704	164	gb|AAO16091.1| pheromone binding protein 3 [Helicoverpa armigera]	3e−106	99%	Yes	Yes
**General odorant binding protein**								
unigene37830	HarmGOBP1	1097	172	emb|CAA65605.1| general odorant binding protein 1 [Heliothis virescens]	4e−101	96%	Yes	Yes
unigene34372	HarmGOBP2	847	162	gb|AAG54078.1| general odorant binding protein 2 [Helicoverpa zea]	3e−113	100%	Yes	Yes
**Other odorant binding protein**								
unigene8328	HarmOBP1	789	147	gb|AEB54580.1| OBP1 [Helicoverpa armigera]	0	100%	Yes	Yes
unigene22398	HarmOBP2	831	143	gb|AEB54586.1| OBP2 [Helicoverpa armigera]	3e−99	99%	Yes	Yes
unigene28809	HarmOBP3	580	147	gb|AEB54582.1| OBP3 [Helicoverpa armigera]	6e−100	99%	Yes	Yes
unigene26687	HarmOBP4	507	137	gb|AEB54584.1| OBP4 [Helicoverpa armigera]	3e−90	98%	No	No
unigene36488	HarmOBP5	924	147	gb|AEB54581.1| OBP5 [Helicoverpa armigera]	1e−99	99%	Yes	Yes
unigene24819	HarmOBP6	572	147	gb|AEB54587.1| OBP6 [Helicoverpa armigera]	9e−90	100%	Yes	Yes
unigene32551	HarmOBP7	570	148	gb|AEB54591.1| OBP7 [Helicoverpa armigera]	2e−80	100%	Yes	Yes
unigene35868	HarmOBP7.2	737	148	gb|AEB54591.1| OBP7 [Helicoverpa armigera]	9e−85	94%	Yes	Yes
unigene6643	HarmOBP8	495	139	gb|AEB54589.1| OBP8 [Helicoverpa armigera]	6e−98	100%	Yes	Yes
unigene8265	HarmOBP9	1655	148	gb|AEB54592.1| OBP9 [Helicoverpa armigera]	1e−101	100%	Yes	Yes
unigene24747	HarmOBP9.2	631	147	gb|AEB54592.1| OBP9 [Helicoverpa armigera]	2e−101	95%	Yes	Yes
unigene37297	HarmOBP13	610	141	gb|AEB54588.1| OBP13 [Helicoverpa armigera]	9e−92	100%	Yes	Yes
unigene15451	HarmOBP14	339	111	gb|ACX53795.1| odorant binding protein [Heliothis virescens]	1e−70	93%	No	No
unigene24215	HarmOBP15	1374	217	gb|ADY17882.1| odorant binding protein [Spodoptera exigua]	4e−75	77%	No	No
unigene28229	HarmOBP16	702	186	gb|ACX53761.1| odorant binding protein [Heliothis virescens]	3e−71	58%	Yes	Yes
unigene24887	HarmOBP17	1184	158	antennal binding protein X [Heliothis virescens]	5e−59	100%	Yes	Yes
unigene4776	HarmOBP18	929	121	gb|AFG72998.1| odorant-binding protein 1 [Cnaphalocrocis medinalis]	8e−70	83%	No	No
unigene16075	HarmOBP19	423	122	ref|NP_001140188.1| odorant-binding protein 4 [Bombyx mori]	2e−29	47%	No	No
unigene23126	HarmOBP20	1100	251	gb|ADD71058.1| odorant-binding protein [Chilo suppressalis]	3e−118	65%	Yes	Yes
unigene34511	HarmOBP21	2207	121	gb|EHJ65654.1| antennal binding protein 4 [Danaus plexippus]	8e−50	66%	No	No
unigene28949	HarmOBP22	1417	121	gb|AFG72998.1| odorant-binding protein 1 [Cnaphalocrocis medinalis]	1e−49	58%	No	No

### Identification of Candidate Chemosensory Proteins

**Figure 5:**
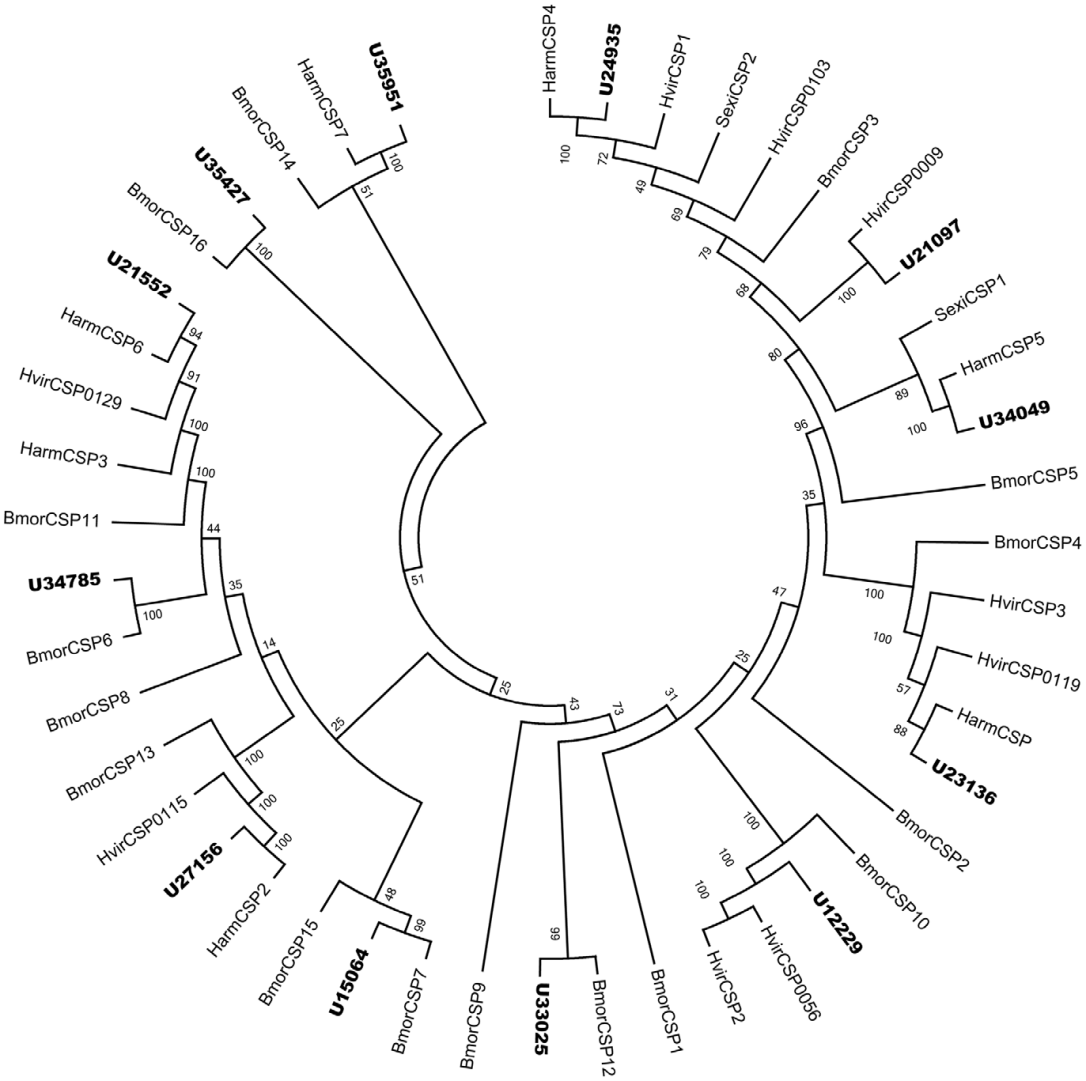
Phylogenetic tree of candidate o chemosensory protein from lepidopterans. Harm: *H. armigera*, Hvir: *H. virescens* ***,*** Bmor: *B. mori*, Se: *S. exigua*, the *H. armigera* unigenes are shown in bold and the letter-unigene in the unigene reference was abbreviated as U.

Bioinformatic analysis led to the identification of 12 different sequences encoding candidate CSPs. Ten sequences were predicted to have full length and all of them had a signal peptide. Neighbor-joining tree showed all the 12 sequences were clustered with one lepidopterans orthologous gene and the candidate CSPs could be well identified (Figure 5). The unigenes corresponding to CSP genes were named following the identified CSPs. The rest 6 CSPs named from HarmCSP8 to HarmCSP13 following the known HarmCSP7. The information of all the 12 CSPs was listed in table 4. The nucleotide sequences of all the 12 CSPs were listed in supplement material S5.

**Table 4:** Unigenes of candidate chemosensory protein.

Unigene reference	Gene name	Length (bp)	ORF (aa)	Blastx best hit (Reference/Name/Species)	E value	Identity	Full length	Signal peptide
**Chemosensory protein**								
unigene23136	HarmCSP	706	127	gb|AAK53762.1|AF368375_1 chemosensory protein [Helicoverpa armigera]	5e−77	100%	Yes	Yes
unigene27156	HarmCSP2	515	120	gb|AEX07265.1| CSP2 [Helicoverpa armigera]	7e−83	100%	Yes	Yes
unigene24935	HarmCSP4	856	128	gb|AEX07269.1| CSP4 [Helicoverpa armigera]	7e−74	100%	Yes	Yes
unigene34049	HarmCSP5	686	127	gb|AEB54579.1| CSP5 [Helicoverpa armigera]	1e−86	100%	Yes	Yes
unigene21552	HarmCSP6	1196	122	gb|AEX07267.1| CSP6 [Helicoverpa armigera]	1e−79	98%	Yes	Yes
unigene35951	HarmCSP7	923	111	gb|AEX07268.1| CSP7 [Helicoverpa armigera]	2e−69	100%	Yes	Yes
unigene21097	HarmCSP8	570	128	gb|ACX53700.1| chemosensory protein [Heliothis virescens]	4e−77	86%	Yes	Yes
unigene12229	HarmCSP9	1084	126	gb|ACX53745.1| chemosensory protein [Heliothis virescens]	8e−72	95%	Yes	Yes
unigene35427	HarmCSP10	926	107	dbj|BAF91720.1| chemosensory protein [Papilio xuthus]	2e−52	94%	Yes	Yes
unigene33025	HarmCSP11	1333	158	gb|EHJ76401.1| chemosensory protein CSP1 [Danaus plexippus]	6e−53	57%	Yes	Yes
unigene15064	HarmCSP12	473	116	gb|ACX53817.1| chemosensory protein [Heliothis virescens]	7e−56	75%	No	No
unigene34785	HarmCSP13	801	122	gb|ACX53719.1| chemosensory protein [Heliothis virescens]	5e−81	98%	Yes	Yes

### Identification of Candidate Sensory Neuron Membrane Proteins

SNMPs were first identified in pheromone-sensitive neurons of Lepidoptera [20] and are thought to play a role in pheromone detection [21]. Two kinds of SNMPs (SNMP1 and SNMP2) have been identified in insects and both kinds of SNMPs were discovered in *H. armigera* transcriptome. The nucleotide sequence of contig5289 was Identical to the HarmSNMP published in Genbank. Contig5355 had 61% identity with SNMP2 of *H. virescens* and was annotation to be SNMP2 of *H. armigera* (Table 5). The nucleotide sequences of the 2 SNMPs were listed in supplement material S5.

**Table 5:** Unigenes of candidate sensory neuron membrane protein.

Unigene reference	Gene name	Length (bp)	ORF (aa)	BLASTx best hit (Reference/Name/Species)	E value	Identity	Full length
**Sensory neuron membrane protein**							
unigene28770	HarmSNMP1	2175	523	gb|AAO15604.1|AF462067_1 sensory neuron membrane protein [Helicoverpa armigera]	0.0	99%	Yes
unigene32002	HarmSNMP2	1727	520	emb|CAP19028.1| sensory neuron membrane protein-2 [Heliothis virescens]	0.0	95%	Yes

### Tissue- and Sex- specific Expression of Candidate *H. armigera* OR, GR and IR Genes

**Figure 6:**
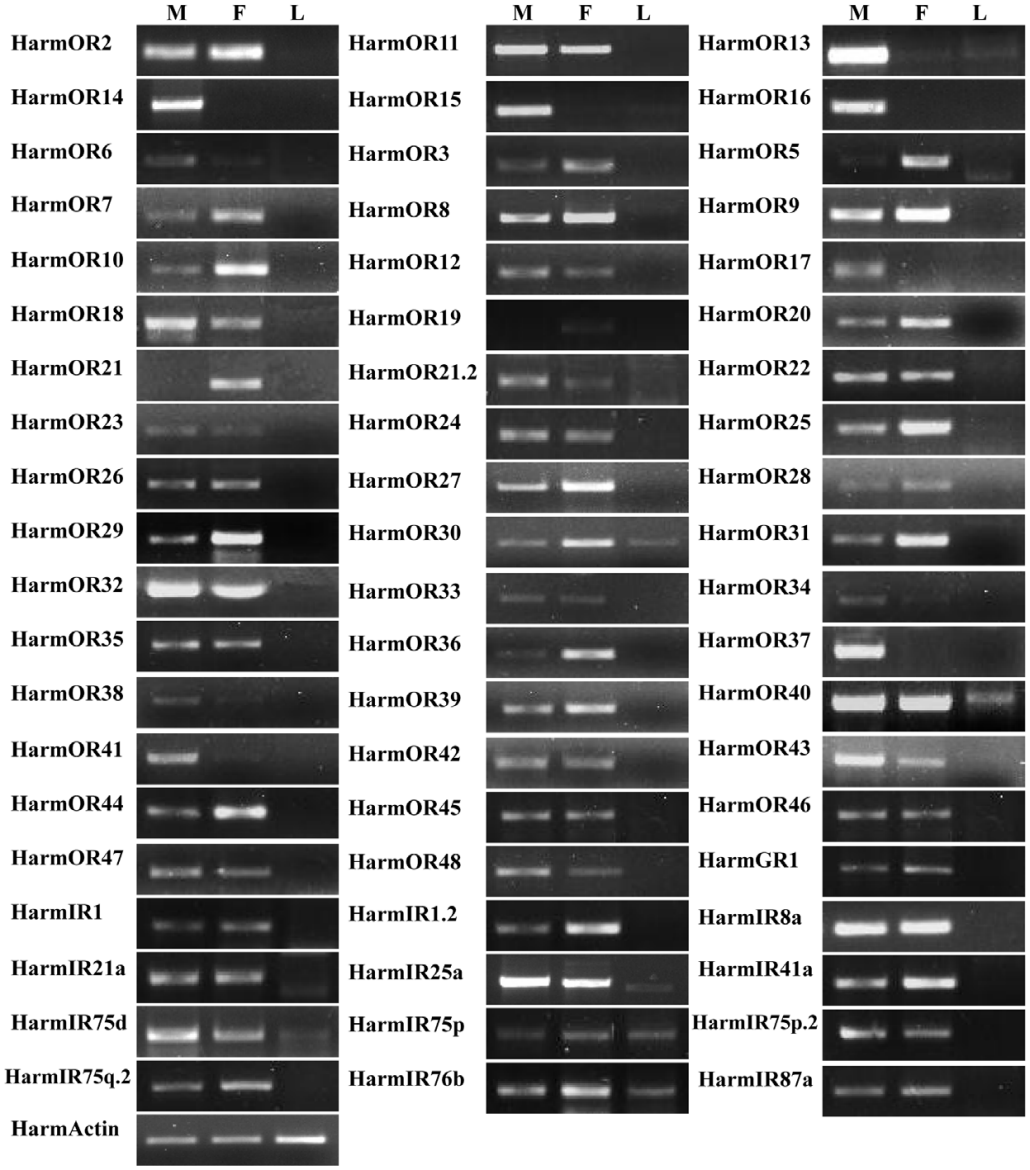
Tissue- and sex- specific expressions of candidates *H. armigera* ORs, GRs and IRs genes. M: male antennae, F: female antennae, L: legs.

The expression patterns of the candidate 47 ORs, 1 GR and 12 IRs in male antennae, female antennae and legs were analyzed by semi-quantitative reverse transcription PCR. Figure 6 shows the detection of all the 60 candidate receptors in antennae of *H. armigera*.

The expressions of all the 47 ORs were detected in antennae. Of the six candidate PRs, four PRs were found to be exclusive to the male antennae and this result was congruent with the expression profile calculated during unigene assembly. The antennal expression level of HarmOR6 was very low. RT-PCR results demonstrated that the ORs HarmOR17, HarmOR37, and HarmOR41 are male-specific; and the ORs HarmOR5and HarmOR21 are female-specific. The remaining 36 ORs were expressed in both sexes, with some of them differentially expressed in male or female antennae. The single GR identified in this study, HarmGR1, was found to be highly expressed at equal levels in antennae of both sexes. Compared to ORs, the expressions of all IRs had no significant difference between males and females.

## Discussion

We used transcriptomic sequencing to identify putative olfactory system genes consisting of 47 ORs, 12 IRs, 26 OBPs, 12 CSPs, and 2 SNMPs in the antennae of *H. armigera*. The olfactory system genes identified in this study are comparable to recently reported insect antennal transcriptome sequence of *M. sexta* with 47 ORs, 6 IRs, 18 OBPs, and 21 CSPs, and *C. pomonella* with 43 ORs and 15 IRs [36], [37]. All the previously annotated or characterized ORs [51], [52], OBPs [53], CSPs [53], and SNMPs of *H. armigera* were included in the candidate olfaction genes identified in this work. Many nonreceptor olfaction genes including OBPs, CSPs and SNMPs were also identified in our antennal transcriptome. The number identified was slightly less than *B. mori*. Probably the remaining OR genes are exclusively expressed in other olfaction organ such as maxillary palp and proboscis or developmental period.

Activated ORs are the first critical step mediating odorant recognition in the peripheral olfactory signal transduction pathway. Only a few OR genes could be identified according to homolog-based strategy on the condition of ORs of closely related species. Previous studies have suggested that single olfactory receptor neuron (ORN) class generally expresses a single OR (except ORCO) [57], and ORN innervates a corresponding glomerulus in the insect olfactory system [57]. While the relationship is not exactly 1∶1:1, the number of glomeruli could form the basis of a rough estimate of the number of ORs in a species [3], [36]. In *H. armigera*, 65 distinct glomeruli have been found in each sex [58] and therefore, the total number of ORs should correspond with the number of glomeruli. Despite much effort searching for ORs in *H. armigera*, only the coreceptor (HaOR2) and ten OR-coding genes or fragments have been identified and deposited in GenBank [51], [52]. In this work, we identified 47 ORs in *H. armigera* antennae and largely extend the number of ORs. Obviously, the number of ORs identified in this study is still less than expected based on the number of glomeruli. There are several possibilities to address the phenomena. Firstly, we only sequenced the antennal transcriptomes of male and female adult. Some OR genes might specifically expressed in different developmental stage such as larval stage or other olfactory organs of adults such as maxillary palp and proboscis [49]. Previous reports indicated that at least 6 ORs and 1o ORs specifically expressed in Bombyx mori and Drosophila larvae antennae, respectively, which supported our hypothesis (Current Biology 2008; Neuron, 2005 Kreher). Secondly, some glomeruli should be innervated by OSNs expressing other classes of chemoreceptors such as ionotropic receptos and gustatory receptors identified in this study. Thirdly, some OR paralogs with highly sequence similarity could be missing in current analysis since they are difficult to be separated with polymorphism without genome sequence(2012 PLOS one Bengtsson). Finally, we couldn’t exclude the possibility that 454 pyrosequencing is not powerful enough to exhaustedly obtain all ORs, especially those ORs with extremely low expression level in the antennae.

Six PRs were identified which were named OR6, OR11, OR13, OR14, OR15, OR16 according *H. virescens* [29]. Only four PRs (OR11, OR13, OR14, and OR16) had been identified in *H. armigera* before. Of the 47 ORs identified in this work, six belong to the PR group. They were orthologous genes of six PRs identified in *H. virescens*. The male-specific or male-enriched expression profiles of these six ORs (Figure 6) were consistent with the PR expression in closely related species, *H. virescens* [29].

All the 41 normal ORs were also specifically expressed in antennae. Experiments were conducted to identify ORs with differential expression patterns between male and female because they might perform specific functions in each gender. The RT-PCR showed that three ORs (HarmOR17, HarmOR37, and HarmOR41) were male specific and two other ORs (HarmOR5 and HarmOR21) were female specific (Figure 6). However, the expression profile calculated according to number of copies found in transcriptomes gave a different result and inconsistencies were also found in other 454 sequences [37]. This may have occurred because 454 reads are suboptimal for expression profiling because the number of reads acquired are relatively low (less than million) compared to Illumina RNA-Seq. In *B. mori*, BmorOR19 and BmorOR30 were found to be specifically expressed in female [49], [59]. We identified one homolog of BmorOR30 (HarmOR48) and two homologs of BmorOR19 (HarmOR21.2 and HarmOR22). But expression of OR48, OR21.2, and OR22 was detected in both male and female *H. armigera* antennae. This may be occurred because the sequences and expression patterns of normal ORs in different insects showed greater specificity than PRs. The sex-specific ORs need further study in *H. armigera*.

Recently, a new family of candidate chemosensory ionotropic receptors was discovered, first in *D. melanogaster* [12] and then in several other species through genome analyses [15]. *D. melanogaster* antennal IRs have been reported to detect a variety of molecules [14]. In *D. melanogaster*, 66 IRs were identified 15 of which proved to be antennae-specific [12], [15]. Twelve IRs were identified in the antennae of *S. littoralis* [35]. We also found 12 IRs in *H. armigera* antennae including two co-receptors, IR8a and IR25a [14]. This is the first report of IRs in *H. armigera*. Sequences alignments showed that the putative *H. armigera* IRs have higher similarity with known IRs than ORs. Unlike ORs, the expression of the IRs appeared to be similar between male and female. Similar results were also observed in the expression study of *S. littoralis* IRs [35]. The relatively high sequence conservation and expression of IRs implies a probable functional conservation. The antennal IRs are a novel group of chemosensory receptors.

### Conclusions

The main objective of antennal transcriptome sequencing was to identify genes potentially involved in olfactory signal detection in *H. armigera*. The numbers of ORs, IRs, OBPS, CSPs and SNMPs identified in this species are close to the complete repertoire of olfactory genes identified from the antennae of other Lepidopteran species. This study demonstrates that high-throughput 454 pyrosequencing enables the recovery of rare or low copy number expressed genes, especially receptor genes with low expression levels in a species without an available genome sequence. Our findings lay the foundation for future research on the molecular basis of olfactory system of *H. armigera* and provide information for comparative and functional genomic analyses of related species. In addition, further studies of olfactory systems, exploring the potential for olfaction-based management of pest moth populations will be feasible with the findings reported in this antennal transcriptome sequencing study.

## Supporting Information

Supplementary Material S1 Accession numbers for amino acid sequences of ORs, IRs, OBPs and CSPs used in phylogenetic analyses.
(DOCX)

Supplementary Material S2 Primers for RT-PCR expression analyses of H. armigera ORs, GR and IRs.
(XLSX)

Supplementary Material S3 Flow chart of RNA sequencing, unigene assemble and functional annotation. The experiment performed in male or female sets was marked in red or green separately. The experiment performed in the blended set of male and female was marked in blue. The numbers without unit in the parentheses were the sequence numbers. The numbers with bp unit in the parentheses were the average base numbers of sequences.
(TIF)

Supplementary Material S4 The gene length, ORF length, expression, GO annotation, and BLAST best hit of each unigene.
(XLSX)

Supplementary Material S5 Unigenes of candidate olfactory genes identified in this study, FASTA formatted file.
(TXT)
