# Supplementary material for: Candidate Olfaction Genes Identified within the Helicoverpa armigera Antennal Transcriptome
Source: PLoS One. 2012 Oct 26;7(10):e48260. doi: 10.1371/journal.pone.0048260 (PMC3482190; doi:10.1371/journal.pone.0048260)
Supplement: Supplementary Material S1 — Accession numbers for amino acid sequences of ORs, IRs, OBPs and CSPs used in phylogenetic analyses. (DOCX) [file pone.0048260.s001.docx]

ORs

| **Protein name** | **Accession number** | **Protein name** | **Accession number** |
| --- | --- | --- | --- |
| HarmOR2 | ADQ13177 | BmorOR17 | BAH66315 |
| HarmOR3 | ACF32961 | BmorOR18 | BAH66316 |
| HarmOR4 | ACF32962 | BmorOR19 | DAA05977 |
| HarmOR9 | ACC63237 | BmorOR20 | BAH66317 |
| HarmOR10 | ACC63238 | BmorOR21 | BAH66318 |
| HarmOR11 | ACF32965 | BmorOR22 | BAH66319 |
| HarmOR12 | ACF32963 | BmorOR23 | BAH66320 |
| HarmOR13 | ACJ12370 | BmorOR24 | BAH66321 |
| HarmOR14 | ACF32964 | BmorOR25 | BAH66322 |
| HarmOR16 | ACS45306 | BmorOR26 | BAH66323 |
| HarmOR18 | ACC63239 | BmorOR27 | BAH66324 |
| HarmOR20 | ACC63240 | BmorOR28 | BAH66325 |
| HvirOR1 | CAD31850 | BmorOR29 | BAH66326 |
| HvirOR2 | CAD31851 | BmorOR30 | BAH66327 |
| HvirOR3 | CAD31852 | BmorOR32 | BAH66328 |
| HvirOR4 | CAD31946 | BmorOR33 | BAH66329 |
| HvirOR5 | CAD31947 | BmorOR34 | BAH66331 |
| HvirOR6 | CAD31948 | BmorOR35 | BAH66332 |
| HvirOR7 | CAD31853 | BmorOR36 | BAH66333 |
| HvirOR8 | CAD31949 | BmorOR37 | BAH66334 |
| HvirOR9 | CAD31950 | BmorOR38 | BAH66335 |
| HvirOR10 | CAG38111 | BmorOR39 | BAH66336 |
| HvirOR11 | CAG38112 | BmorOR40 | BAH66337 |
| HvirOR12 | CAG38113 | BmorOR41 | DAA05997 |
| HvirOR13 | CAG38114 | BmorOR42 | BAH66338 |
| HvirOR14 | CAG38115 | BmorOR44 | BAH66339 |
| HvirOR15 | CAG38116 | BmorOR45 | BAH66340 |
| HvirOR16 | CAG38117 | BmorOR46 | BAH66341 |
| HvirOR17 | CAG38118 | BmorOR47 | BAH66342 |
| HvirOR18 | CAG38119 | BmorOR49 | BAH66343 |
| HvirOR19 | CAG38120 | BmorOR50 | BAH66345 |
| HvirOR20 | CAG38121 | BmorOR51 | BAH66346 |
| HvirOR21 | CAG38122 | BmorOR53 | BAH66347 |
| BmorOR1 | BAD69584 | BmorOR54 | BAH66348 |
| BmorOR2 | BAD69585 | BmorOR55 | BAH66349 |
| BmorOR3 | BAD89567 | BmorOR56 | BAH66350 |
| BmorOR4 | BAD89568 | BmorOR57 | BAH66351 |
| BmorOR5 | BAD89569 | BmorOR58 | BAH66352 |
| BmorOR6 | BAD89570 | BmorOR59 | BAH66353 |
| BmorOR7 | NP_001106227 | BmorOR60 | BAH66354 |
| BmorOR8 | BAH66308 | BmorOR61 | BAH66355 |
| BmorOR9 | BAH66309 | BmorOR62 | BAH66357 |
| BmorOR10 | DAA05970 | BmorOR63 | BAH66358 |
| BmorOR11 | BAH66310 | BmorOR64 | BAH66359 |
| BmorOR12 | BAH66311 | BmorOR65 | BAH66360 |
| BmorOR13 | BAH66312 | BmorOR66 | BAH66361 |
| BmorOR14 | BAH66313 | BmorOR67 | BAH66362 |
| BmorOR15 | DAA05974 | BmorOR68 | BAH66363 |
| BmorOR16 | BAH66314 |  |  |

IRs

| **Protein name** | **Accession number** | **Protein name** | **Accession number** |
| --- | --- | --- | --- |
| SlitIR21a | ADR64678 | DmelIR7d | Croset et al., 2010 |
| SlitIR25a | ADR64679 | DmelIR7e | Croset et al., 2010 |
| SlitIR40a | ADR64680 | DmelIR7f | Croset et al., 2010 |
| SlitIR41a | ADR64681 | DmelIR7g | Croset et al., 2010 |
| SlitIR68a | ADR64682 | DmelIR10a | Croset et al., 2010 |
| SlitIR75d | ADR64683 | DmelIR11a | Croset et al., 2010 |
| SlitIR75p | ADR64684 | DmelIR20a | Croset et al., 2010 |
| SlitIR75q.2 | ADR64685 | DmelIR41a | Croset et al., 2010 |
| SlitIR75q.1 | ADR64686 | DmelIR47a | Croset et al., 2010 |
| SlitIR76b | ADR64687 | DmelIR47b | Croset et al., 2010 |
| SlitIR1 | ADR64688 | DmelIR48a | Croset et al., 2010 |
| SlitIR87a | ADR64689 | DmelIR48b | Croset et al., 2010 |
| BmorIR87a | Croset et al., 2010 | DmelIR48c | Croset et al., 2010 |
| BmorIR7d.3 | Croset et al., 2010 | DmelIR51a | Croset et al., 2010 |
| BmorIR7d.1 | Croset et al., 2010 | DmelIR51b | Croset et al., 2010 |
| BmorIR7d.2 | Croset et al., 2010 | DmelIR52a | Croset et al., 2010 |
| BmorIR143 | Croset et al., 2010 | DmelIR52b | Croset et al., 2010 |
| BmorIR68a | Croset et al., 2010 | DmelIR52c | Croset et al., 2010 |
| BmorIR41a | Croset et al., 2010 | DmelIR52d | Croset et al., 2010 |
| BmorIR21a | Croset et al., 2010 | DmelIR54a | Croset et al., 2010 |
| BmorIR64a | Croset et al., 2010 | DmelIR56a | Croset et al., 2010 |
| BmorIR75d | Croset et al., 2010 | DmelIR56b | Croset et al., 2010 |
| BmorIR75p | Croset et al., 2010 | DmelIR56c | Croset et al., 2010 |
| BmorIR75q.1 | Croset et al., 2010 | DmelIR56d | Croset et al., 2010 |
| BmorIR75q.2 | Croset et al., 2010 | DmelIR56e | Croset et al., 2010 |
| BmorIR40a | Croset et al., 2010 | DmelIR60a | Croset et al., 2010 |
| BmorIR76b | Croset et al., 2010 | DmelIR60b | Croset et al., 2010 |
| BmorIR93a | Croset et al., 2010 | DmelIR60c | Croset et al., 2010 |
| BmorIR8a | Croset et al., 2010 | DmelIR60d | Croset et al., 2010 |
| BmorIR25a | Croset et al., 2010 | DmelIR60e | Croset et al., 2010 |
| DmelIR8a | Croset et al., 2010 | DmelIR60f | Croset et al., 2010 |
| DmelIR25a | Croset et al., 2010 | DmelIR62a | Croset et al., 2010 |
| DmelIR21a | Croset et al., 2010 | DmelIR67a | Croset et al., 2010 |
| DmelIR31a | Croset et al., 2010 | DmelIR67b | Croset et al., 2010 |
| DmelIR40a | Croset et al., 2010 | DmelIR67c | Croset et al., 2010 |
| DmelIR64a | Croset et al., 2010 | DmelIR68a | Croset et al., 2010 |
| DmelIR75a | Croset et al., 2010 | DmelIR68b | Croset et al., 2010 |
| DmelIR75b | Croset et al., 2010 | DmelIR85a | Croset et al., 2010 |
| DmelIR75c | Croset et al., 2010 | DmelIR87a | Croset et al., 2010 |
| DmelIR75d | Croset et al., 2010 | DmelIR94a | Croset et al., 2010 |
| DmelIR76a | Croset et al., 2010 | DmelIR94b | Croset et al., 2010 |
| DmelIR76b | Croset et al., 2010 | DmelIR94c | Croset et al., 2010 |
| DmelIR84a | Croset et al., 2010 | DmelIR94d | Croset et al., 2010 |
| DmelIR92a | Croset et al., 2010 | DmelIR94e | Croset et al., 2010 |
| DmelIR93a | Croset et al., 2010 | DmelIR94f | Croset et al., 2010 |
| DmelIR7a | Croset et al., 2010 | DmelIR94g | Croset et al., 2010 |
| DmelIR7b | Croset et al., 2010 | DmelIR94h | Croset et al., 2010 |
| DmelIR7c | Croset et al., 2010 | DmelIR100a | Croset et al., 2010 |

OBPs

| **Protein name** | **Accession number** | **Protein name** | **Accession number** |
| --- | --- | --- | --- |
| HarmPBP1 | AEB54585 | BmorOBP3 | Gong et al. 2009 |
| HarmPBP2 | AEB54583 | BmorOBP4 | Gong et al. 2009 |
| HarmPBP3 | AAO16091 | BmorOBP6 | Gong et al. 2009 |
| HarmGOBP1 | AAL09821 | BmorOBP7 | Gong et al. 2009 |
| HarmGOBP2 | CAC08211 | BmorOBP8 | Gong et al. 2009 |
| HarmOBP1 | AEB54580 | BmorOBP11 | Gong et al. 2009 |
| HarmOBP2 | AEB54586 | BmorOBP13 | Gong et al. 2009 |
| HarmOBP3 | AEB54582 | BmorOBP15 | Gong et al. 2009 |
| HarmOBP4 | AEB54584 | BmorOBP17 | Gong et al. 2009 |
| HarmOBP5 | AEB54581 | BmorOBP18 | Gong et al. 2009 |
| HarmOBP6 | AEB54587 | BmorOBP20 | Gong et al. 2009 |
| HarmOBP7 | AEB54591 | BmorOBP21 | Gong et al. 2009 |
| HarmOBP8 | AEB54589 | BmorOBP22 | Gong et al. 2009 |
| HarmOBP9 | AEB54592 | BmorOBP23 | Gong et al. 2009 |
| HarmOBP13 | AEB54588 | BmorOBP24 | Gong et al. 2009 |
| HvirPBP1 | CAA65604 | BmorOBP25 | Gong et al. 2009 |
| HvirPBP2 | CAL48346 | BmorOBP26 | Gong et al. 2009 |
| HvirGOBP1 | CAA65605 | BmorOBP27 | Gong et al. 2009 |
| HvirGOBP2 | CAA65606 | BmorOBP29 | Gong et al. 2009 |
| HvirABP0107 | ACX53792 | BmorOBP30 | Gong et al. 2009 |
| HvirOBP0021 | ACX53711 | BmorOBP31 | Gong et al. 2009 |
| HvirPBP0046 | ACX53735 | BmorOBP32 | Gong et al. 2009 |
| HvirOBP0005 | ACX53696 | BmorOBP33 | Gong et al. 2009 |
| HvirABP0112 | ACX53797 | BmorOBP34 | Gong et al. 2009 |
| HvirOBP0136 | ACX53819 | BmorOBP36 | Gong et al. 2009 |
| HvirOBP0058 | ACX53747 | BmorOBP37 | Gong et al. 2009 |
| HvirABP2 | CAC33574 | BmorOBP38 | Gong et al. 2009 |
| HvirOBP0110 | ACX53795 | BmorOBP39 | Gong et al. 2009 |
| HvirOBP0072 | ACX53761 | BmorOBP40 | Gong et al. 2009 |
| HvirOBP0054 | ACX53743 | BmorOBP41 | Gong et al. 2009 |
| HvirOBP0067 | ACX53756 | BmorOBP42 | Gong et al. 2009 |
| HvirABPX | CAA05508 | BmorOBP43 | Gong et al. 2009 |
| BmorOBP1 | Gong et al. 2009 | BmorOBP44 | Gong et al. 2009 |
| BmorOBP2 | Gong et al. 2009 |  |  |

CSPs

| **Protein name** | **Accession number** | **Protein name** | **Accession number** |
| --- | --- | --- | --- |
| HarmCSP | AAK53762 | BmorCSP1 | ABH88194 |
| HarmCSP2 | AEX07265 | BmorCSP2 | ABH88195 |
| HarmCSP3 | AEX07266 | BmorCSP3 | ABH88196 |
| HarmCSP4 | AEX07269 | BmorCSP4 | ABH88197 |
| HarmCSP5 | AEB54579 | BmorCSP5 | ABH88198 |
| HarmCSP6 | AEX07267 | BmorCSP6 | ABH88199 |
| HarmCSP7 | AEX07268 | BmorCSP7 | ABH88200 |
| HvirCSP1 | AAM77041 | BmorCSP8 | ABH88201 |
| HvirCSP2 | AAM77040 | BmorCSP9 | ABH88202 |
| HvirCSP3 | AAM77042 | BmorCSP10 | ABH88203 |
| HvirCSP0009 | ACX53700 | BmorCSP11 | ABH88204 |
| HvirCSP0103 | ACX53788 | BmorCSP12 | ABH88205 |
| HvirCSP0129 | ACX53813 | BmorCSP13 | ABH88206 |
| HvirCSP0056 | ACX53745 | BmorCSP14 | ABH88207 |
| HvirCSP0119 | ACX53804 | BmorCSP15 | ABH88208 |
| HvirCSP0115 | ACX53800 | BmorCSP16 | ABH88209 |
| SexiCSP1 | ABM67688 |  |  |
| SexiCSP2 | ABM67689 |  |  |
