# Supplementary figures and images for: Candidate Olfaction Genes Identified within the Helicoverpa armigera Antennal Transcriptome
Source: PLoS One. 2012 Oct 26;7(10):e48260. doi: 10.1371/journal.pone.0048260 (PMC3482190; doi:10.1371/journal.pone.0048260)

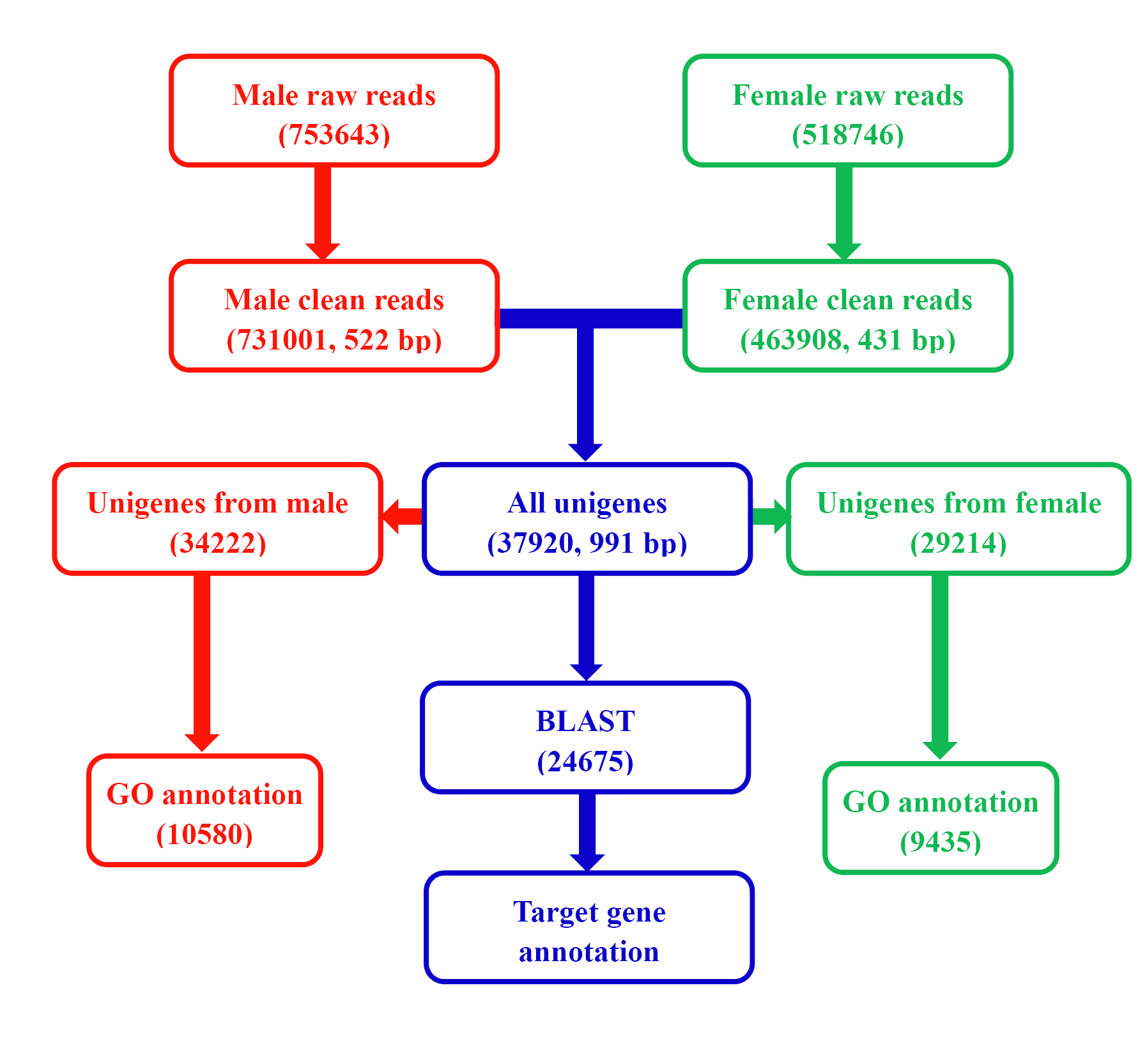

Supplement: Supplementary Material S3 — Flow chart of RNA sequencing, unigene assemble and functional annotation. The experiment performed in male or female sets was marked in red or green separately. The experiment performed in the blended set of male and female was marked in blue. The numbers without unit in the parentheses were the sequence numbers. The numbers with bp unit in the parentheses were the average base numbers of sequences. (TIF) [file pone.0048260.s003.tif]
